# Utilizing Artificial Intelligence for Microbiome Decision-Making: Autism Spectrum Disorder in Children from Bosnia and Herzegovina

**DOI:** 10.3390/diagnostics14222536

**Published:** 2024-11-13

**Authors:** Džana Bašić-Čičak, Jasminka Hasić Telalović, Lejla Pašić

**Affiliations:** 1 Computer Science Department, University Sarajevo School of Science and Technology, Hrasnička cesta 3a, 71000 Sarajevo, Bosnia and Herzegovina; dzana.basic@stu.ssst.edu.ba; 2 Sarajevo Medical School, University Sarajevo School of Science and Technology, Hrasnička cesta 3a, 71000 Sarajevo, Bosnia and Herzegovina; lejla.pasic@ssst.edu.ba

**Keywords:** gut microbiome, ASD, artificial intelligence, machine learning, 16S rRNA

## Abstract

Background/Objectives: The study of microbiome composition shows positive indications for application in the diagnosis and treatment of many conditions and diseases. One such condition is autism spectrum disorder (ASD). We aimed to analyze gut microbiome samples from children in Bosnia and Herzegovina to identify microbial differences between neurotypical children and those with ASD. Additionally, we developed machine learning classifiers to differentiate between the two groups using microbial abundance and predicted functional pathways. Methods: A total of 60 gut microbiome samples (16S rRNA sequences) were analyzed, with 44 from children with ASD and 16 from neurotypical children. Four machine learning algorithms (Random Forest, Support Vector Classification, Gradient Boosting, and Extremely Randomized Tree Classifier) were applied to create eight classification models based on bacterial abundance at the genus level and KEGG pathways. Model accuracy was evaluated, and an external dataset was introduced to test model generalizability. Results: The highest classification accuracy (80%) was achieved with Random Forest and Extremely Randomized Tree Classifier using genus-level taxa. The Random Forest model also performed well (78%) with KEGG pathways. When tested on an independent dataset, the model maintained high accuracy (79%), confirming its generalizability. Conclusions: This study identified significant microbial differences between neurotypical children and children with ASD. Machine learning classifiers, particularly Random Forest and Extremely Randomized Tree Classifier, achieved strong accuracy. Validation with external data demonstrated that the models could generalize across different datasets, highlighting their potential use.

## 1. Introduction

The development of novel sequencing technologies over the past two decades resulted in the generation of many microbiome datasets that correspond to human health and disease states. Such datasets were initially analyzed solely using statistics to infer population information. More recently, machine learning techniques, as an exemplary branch of artificial intelligence algorithms, have been deployed to aid medical decision-making.

Machine learning models have been developed with gut microbiome data aiming to enhance screening and diagnostic methods. A collection of various studies in this field is outlined hereafter. Most of these studies employed OTUs as characteristics, while one study supplemented this approach by incorporating k-mers.

Pasolli et al. [1] scrutinized six datasets representing five conditions: liver cirrhosis, colorectal cancer, IBD, obesity, and type II diabetes mellitus. They assessed four distinct machine learning algorithms—Random Forest, Support Vector Machines (SVMs), elastic net, and LASSO—and determined their strong disease-prediction capabilities. Reiman et al. [2] applied a convolutional neural network (CNN), a deep learning algorithm, to analyze datasets sourced from three bodily locations, including the gut. The CNN demonstrated potential in accurately classifying the body site origin of diverse metagenomes. In a separate investigation, Ai et al. [3] explored colorectal cancer prediction based on gut microbiota using Bayesian networks, Random Forest, and logistic algorithms. They observed that these predictions outperformed the standard fecal occult blood diagnostic test. Asgari et al. [4] explored a combination of deep learning methods (Multi-Layer-Perceptron neural network) in addition to SVM and Random Forest to identify microbiomes in terms of body site origin and the presence of Crohn’s disease. They found that the usage of k-mers outperformed the OTU approach.

Applying machine learning to medical decision-making has demonstrated potential in environments of complex etiology, formed as a combination of environmental and genetic aspects, such as obesity, diabetes mellitus, irritable bowel disease, Crohn’s disease, colorectal cancer, and multiple sclerosis [5,6].

Several scholarly inquiries have scrutinized the utilization of machine (including deep) learning methodologies in the analysis of microbiome data [5,7,8,9,10,11]. These investigations have delineated the potential applications of such methodologies and guidelines for best practices in undertaking research endeavors in this domain. Our methodological framework for the present project has been informed by insights gleaned from these seminal works.

Autism spectrum disorder (ASD) is a medical condition, a neurodevelopmental and neuropsychiatric disorder characterized by a young age at onset, and recognized by deficits in social functioning, cognitive inflexibility, and repetitive behaviors [12]. An increase in ASD awareness, detection, diagnosis, prognosis, and treatment occurred in the last two decades and, currently, ASD’s global prevalence estimate is over 1% [13].

ASD’s pathophysiology is a result of genetic and environmental factors’ interactions. Several hundreds of genes have been identified as involved in ASD onset so far, and a number of those regulate synaptic function [14]. An increased risk of ASD development was associated with mutations and duplications of these genes, as well as the presence of single-nucleotide polymorphisms, and/or epigenetic changes with an effect on gene activity and expression [15]. It was estimated that the inheritable nature of familial risk for ASD ranges from 50 to 83% [16].

The nature of environmental factors implicated in ASD development is equally diverse and contributes to about 40–50% of the variance. It includes parental age, delivery option, birth conditions, interpregnancy interval, birth parity and order, gestational age, infections, maternal use of medications, heavy metal exposure, air pollution, and environmental toxins [17].

Several medical comorbidities have been associated with ASD and they involve immune dysregulation and a higher prevalence of general gastrointestinal problems (up to four times) [18].

A significant challenge in ASD lies within its diagnosis, which remains complex. Current diagnostic tools necessitate children to exhibit deficiencies in social–emotional reciprocity, nonverbal communicative behaviors employed for social interaction, and difficulties in establishing, maintaining, and comprehending relationships. These criteria are evaluated using various screening questionnaires, yet they can be influenced by biases, especially concerning sex/gender differences in clinical presentation [19]. Moreover, these evaluation procedures involve prolonged observations, leading to considerable delays between the observed onset of the disorder and its formal diagnosis.

The gut microbiome, a collection of microorganisms within the digestive system, collectively governs intestinal physiology and its linked immune function [20]. Over the last decade, gut microbiome (both 16S rRNA and shotgun) sequences have been analyzed and revealed that individuals diagnosed with ASD exhibit variations in gut microbiota compared to neurotypical individuals. Early studies reported variances including a reduced Bacteroidetes/Firmicutes ratio, diminished *Prevotella* levels, and elevated presence of *Suterella*, *Lactobacillus*, and *Desulfovibrio* [20].

In the last couple of years, several studies have examined the microbiome data of children with ASD and occasionally supplemented the 16S rRNA data with a machine learning approach. Vernocchi et al. [21] analyzed the gut microbiota of 41 children with ASD and 35 age-matched (3–15 years) neurotypical children located in Italy. Quadratic Discriminant Analysis and Gaussian Process Classifier machine learning models were used to test the classification based on operational taxonomic units (OTUs) and KEGG ortholog pathways. They reported an overall classification accuracy of 80% in the distinction between ASD and control patients based on OTUs and 73% accuracy based on the KEGG pathways. Son et al. [22] assessed associations between ASD phenotype, functional GI disorders, and fecal microbiota in simplex families, which had only a single ASD proband and neurotypical (NT) siblings. In total, 66 ASD probands and 37 NT siblings were recruited in Canada and the United States. No significant difference in diversity or overall microbial composition was detected between children with ASD and their NT siblings. Exploratory analysis of the 16S rRNA sequencing data, however, identified several low-abundance taxa binned at the genus level that were associated with ASD: *Chloroplast*, *Asteroleplasma*, *Thalassospira*, *Burkholderia*, *Comamonadaceae*, *Fusobacteriales*, *Prevotellaceae*, and *Mobiluncus.* This and the following study did not employ machine learning approaches.

In their 2018 study, Coretti et al. [23] examined the gut microbiota composition and levels of fecal short-chain fatty acids in a group of Italian children aged 2–4 years with autism spectrum disorder (ASD), comparing the findings to those of neurotypical controls. The results showed a notable increase in Bacteroidetes and Proteobacteria populations, alongside a significant reduction in Actinobacteria, within the ASD group. Among the 91 operational taxonomic units (OTUs) that were differentially abundant between these two groups, the researchers observed a pronounced reduction in *Bifidobacterium longum* and an elevated abundance of *Faecalibacterium prausnitzii* in ASD-affected children. These findings suggest potential microbiome dysbiosis in ASD and highlight specific bacterial taxa that may play critical roles in the gut–brain axis.

Dan et al. [23] investigated the gut microbial population in a cohort of 143 children aged 2–13 years old. The authors reported that the α-diversity of the ASD group showed no significant change with age, while the typically developing (TD) group showed increased α-diversity with age, indicating that the compositional development of the gut microbiota in ASD varies at different ages in ways that are not consistent with the TD group. A Random Forest model trained on 16S rRNA abundance profiles with key genera as input variables demonstrated strong discriminatory power in predicting ASD status through a nested 10-fold cross-validation process.

Pulikkan et al. [24] compared the fecal microbiota of 30 children with ASD with 24 family-matched healthy children from an Indian population. The authors identified differentiating OTUs using the Random Forest approach, which has shown prominent dysbiosis in the gut microbiome of children with ASD, with higher relative abundances of families Lactobacillaceae, Bifidobacteraceae, and Veillonellaceae, whereas the gut microbiome of healthy children was dominated by the family Prevotellaceae.

Nonetheless, there has not been consistent agreement in findings due to studies being conducted across diverse geographical regions and the microbial makeup of the human gut being influenced by numerous factors such as cultural practices and dietary habits [20].

As a result of the above studies, probiotic therapies and fecal transplants have arisen as promising new treatments for enhancing gastrointestinal and behavioral issues in individuals with ASD. A recent report highlighted that fecal transplantation resulted in a sustained improvement of children’s symptoms, lasting for two years following the conclusion of treatment [25].

Detecting ASD at an early stage is linked to more favorable outcomes due to the opportunity for early intervention. A recent review, encompassing fourteen studies, highlighted the largely positive impact of early intervention approaches on ASD outcomes. Furthermore, the universally recognized benefit of these interventions lies in their provision of support services to caregivers [26].

In this study, we have analyzed the gut microbiome dataset of neurotypical children and children with ASD from Bosnia and Herzegovina. To the best of our knowledge, this is the first human microbiome analysis from Bosnia and Herzegovina. We deployed a bioinformatic pipeline for the OTUs’ detection and statistical analysis to identify the difference between the two groups within the dataset. The list of acquired OTUs was utilized as input features for the construction of a machine learning classifier capable of confidently detecting whether an unknown sample originated from a neurotypical child or a child with ASD.

## 2. Materials and Methods

### 2.1. Dataset Generation

The participants were 44 children with ASD (diagnosis code F84.0 according to ICD-10 classification) and 16 children without ASD diagnosis (control group). We recruited children of both sexes, ages 3–12 years. Exclusion criteria for the ASD group were the absence of adequate diagnosis and use of antibiotics, prebiotics, or probiotics within six months prior to the start of the study. The exclusion criterion for the control group was the use of antibiotics, prebiotics, or probiotics within six months prior to the start of the study. The study was approved by the Ethic Committee of the University Sarajevo School of Science and Technology (approval No. 004-Ethicalout/2020). Written informed consent was obtained from the parents/guardians of study participants.

Recruitment occurred from September 2020 to May 2021. The average age of the participants was 6.2 years in the ASD group and 5.7 years in the control group. The sex ratio in the ASD group was 1:5 (16% girls and 84% boys) and 1:4 in the control group (25% girls and 75% boys).

Parents/guardians of the enrolled children collected morning stool samples using an OMNIgene^®^-GUT OM-200 kit (DNA Genotek, Ottawa, ON, Canada) according to the manufacturer’s instructions. These samples were kept at room temperature and analyzed within one week of collection. The total community DNA was isolated using a QiaAmp PowerFecal Pro DNA kit (Qiagen, Hilden, Germany) according to the manufacturer’s instructions. Extracted DNAs were checked for quality and quantity by fluorometric measurements with Qubit^®^ fluorometer (Thermo Fisher Scientific Inc., Waltham, MA, USA). The obtained concentrations ranged from 50 to 200 ng/μL. The DNAs were stored at −20 °C until further processing.

Sequencing was conducted by Alea Genetički centar (Sarajevo, Bosnia and Herzegovina) as described below. Sequencing samples were prepared using the 16S Metagenomic Sequencing Library Preparation protocol for the Illumina MiSeq™ System (Illumina, San Diego, CA, USA). We conducted amplicon PCR using V3-V4-region-specific forward primer 5′-TCGTCGGCAGCGTCAGATGTGTATAAGAGACAGCCTACGGGNGGCWGCAG and reverse primer 5′- GTCTCGTGGGCTCGGAGATGTGTATAAGAGACAGGACTACHVGGGTATCTAATCC (Klindworth et al., 2013) [27] as well as PrimeSTAR^®^ Max DNA polymerase (Takara Bio Inc., Kusatsu, Japan). Amplified regions were then indexed using IDT^®^ indexes (Illumina, San Diego, CA, USA) and PrimeSTAR^®^ Max DNA polymerase (Takara Bio Inc., Kusatsu, Japan) according to the manufacturer’s protocol. We purified both types of amplicons after PCR using Agencourt AMPure XP beads (Beckman Coulter Inc., Brea, CA, USA). We determined the sizes of amplified fragments using an Agilent 2100 Bioanalyzer (Agilent Technologies, Santa Clara, CA, USA) and concentrations of amplified DNA using a Qubit^®^ fluorometer (Thermo Fisher Scientific Inc., Waltham, MA, USA). We then pooled the normalized libraries, denatured them with NaOH, and combined them with 25% (*v*/*v*) denatured 10 pM PhiX as instructed by the manufacturer. Sequencing was performed on the Illumina MiSeq sequencing system using v3 reagent kit (Illumina, San Diego, CA, USA). To avoid the ‘batch effect’, we analyzed all samples in a single batch.

### 2.2. Validation Datase

For validation purposes, we introduced an additional dataset originating from a study by Coretti et al. [28] as we aimed to test whether the computational model is dataset-specific or is also successful in the classification of additional independent datasets. The validation dataset consisted of 11 samples of individuals with ASD and 14 samples labeled as healthy controls. The V3–V4 16S rRNA gene region was sequenced to discover the taxonomy of the samples. All the subjects were between 2 and 4 years old, with male samples being predominant (68%).

### 2.3. Bioinformatic Analysis

Filtering high-quality reads, adeptly trimming adapters, and denoising reads to generate amplicon sequence variants (ASVs) were performed using DADA2 within the QIIME2-2023.7 platform [29].

Taxonomic classification was executed using the classify-sklearn method and SILVA reference database [30]. Before classification, the SILVA database underwent preprocessing through RESCRIPt [31]. Employing a 99% sequence similarity threshold, sequences were organized into operational taxonomic units (OTUs).

QIIME2 was used to generate bar plots, PCoA (principal coordinate analysis) plots, and taxonomic summaries and to estimate measures of alpha- and beta-diversity. To facilitate further analysis (outside of QIIME2), the absolute abundances of bacteria were normalized using a custom Python version 3.0 script.

Predictive functional analysis was performed using the 16S rRNA marker gene in conjunction with a reference genome database, facilitated by the q2-PICRUSt computational tool (Douglas et al., 2020 [32]).

To complement our functional analysis, we integrated KEGG descriptions from the online database developed by Kanehisa and Goto [33].

### 2.4. Machine Learning

The performance of four different ML algorithms was investigated on a dataset. The tested algorithms were Random Forest (RF), Support Vector Machine (SVM), Gradient Boosting (GB), and Extremely Randomized Tree Classifier, as described by Telalović et al. [34]. The classification was tested using taxonomy, as well as the functional pathway data, as data features. The original dataset was imbalanced, so we applied the Synthetic Minority Oversampling Technique (SMOTE) to create new data points for the minority class and thus balance the dataset. Otherwise, a trivial algorithm that always predicts that the sample came from the majority group would have very significant accuracy (73%) but problems would be discovered when examining specificity and sensitivity metrics. Random oversampling of the minority class could introduce overfitting of the model. The SMOTE function creates new observations by interpolating randomly chosen points and their nearest neighbors, thus reducing the danger of overfitting. To evaluate the model performance, three metrics were reported: accuracy, sensitivity, and specificity. Accuracy is defined as the portion of the correctly classified samples overall, while sensitivity is the portion of correctly classified positive (ASD) samples and specificity is the portion of correctly classified negative (control) samples.

Feature importance was calculated based on the scikit-learn built-in feature importance method. A Random Forest classification model was developed, and the importance was computed as the mean and standard deviation of accumulation of the impurity decrease within each tree. The way this method works is that during the construction of decision trees, the algorithm selects the best feature to split based on the criterion that maximizes the decrease in impurity. In our case, after the Random Forest model was built, the decrease in impurity that each feature provided at each node was accumulated, and the average decrease across all trees was computed. The idea is that when a feature is used to split a node, it contributes to making the resulting child nodes more homogenous. The same algorithm can be used on other classification models built, but the decision to use Random Forest was based on multiple sources, including Marcos-Zambrano et al. [7] and Pulikkan et al. [24].

Prior to the final classification, all the hyper-parameters of the estimators were tuned. This was carried out by using the GridSearchCV function available in the sklearn package [35]. This function exhaustively searches the list of specified parameter values in order to return the ones that lead to the highest accuracies.

**Figure 1:**
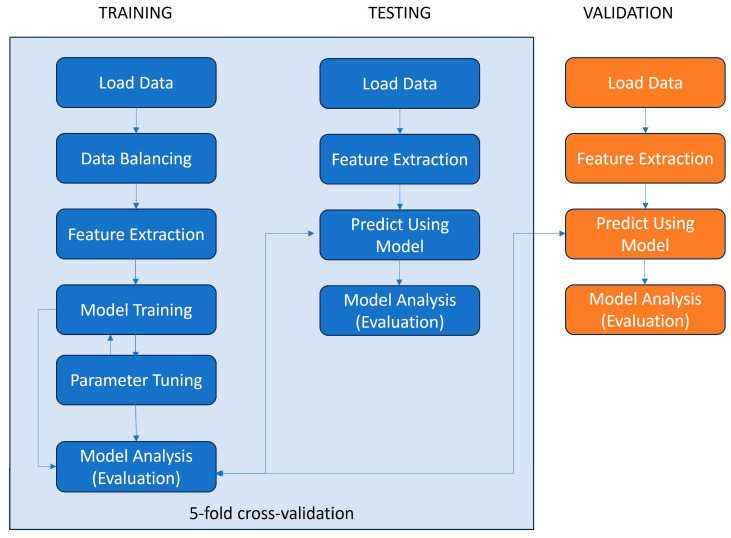
Data processing pipeline for ML model training, testing, and validation.

The classification model was trained and tested (using 5-fold cross-validation) on the dataset generated for this paper and it was additionally validated using an independent dataset [28]. The steps of the machine learning pipeline deployed for this paper are depicted in Figure 1.

### 2.5. Statistical Analysis

For the statistical description of the dataset, average values of relative abundances in the dataset were calculated and represented as a percentage of the total. Mean values were reported on phylum and genus levels. The ten most abundant genera in both groups were reported. To assess the statistical difference in relative abundances between the ASD and control groups, the Mann–Whitney U-test was calculated using the mannwhitneyu() function from the scipy.stats library in Python.

Alpha-diversity was determined using Shannon, Evenness, and observed species diversity indices, and the *p* value for group comparisons was determined by Kruskal–Wallis’s test [36].

Principal coordinate analyses (PCoA) plots were constructed to illustrate the beta-diversity of samples based on Phylogenetically informed weighted and unweighted Unifrac [37] and Bray–Curtis’s metrics. To test the association between the covariates and beta-diversity measures, permutational analysis of variance (PERMANOVA) was used. Entire α- and β-diversity visualizations and statistical calculations were carried out using the QIIME2-2023.7 tool.

## 3. Results

### 3.1. Dataset Characteristics

Table 1 summarizes the metadata obtained in the analyzed dataset. The mean age was 6.10 years in the ASD group and 5.25 in the control group. Both groups were dominated by male subjects, but this ratio was higher in the ASD group (5.28 versus 3.00). Almost all children from the ASD group (90%) reported the presence of at least one gastrointestinal disorder symptom, while this frequency was lower in the control group (12.5%). Finally, all children from the ASD group had speech disorders, compared to 12.5% in the control group.

### 3.2. Statistical Analysis of the Dataset

The demultiplexed sequence summary was as follows: a total of 1,216,304 sequence reads of 16S rRNA gene amplicons were obtained with an average of 20,271 reads/sample and an average length of 300 base pairs. The number of taxa identified for three study groups at the five different taxonomic levels is depicted in Table 2. The ASD group had a higher number of taxa at each taxonomic level.

At the phylum level, seven phyla (Firmicutes, Bacteroidetes, Proteobacteria, Actinobacteria, Verrucomicrobia, Cyanobacteria, and Euryarchaeota) were detected but no statistically significant differences in phylum abundances were found. We calculated the Firmicutes/Bacteroidetes ratio for both datasets. This ratio was higher in the ASD group (2.25) than in the control group (2.03) but the difference was not statistically significant (*p* = 0.68, Mann–Whitney U Test).

At the genus level, only the abundance of *Streptococcus* was significantly different between the ASD and control groups (*p* = 0.00054, Mann–Whitney U Test) with it being less abundant in the ASD group.

**Figure 2:**
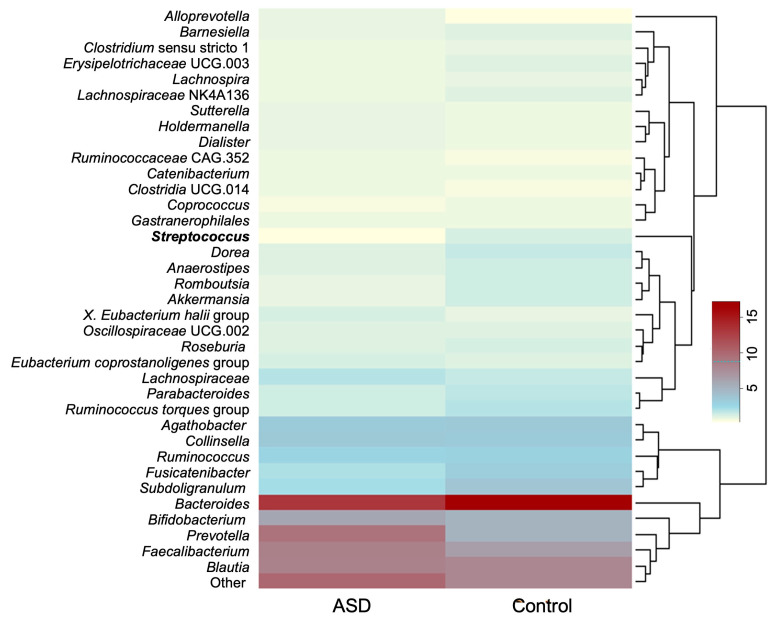
Average relative abundance of most abundant bacterial genera (relative abundance > 0.1%). Genus whose relative abundance differs significantly between the datasets is presented in bold. Legend: ASD—autism spectrum disorder group; control—control group).

The average relative abundances of genera whose relative abundance was >1.0% in the dataset are presented in Figure 2. The ten most abundant genera discovered in both groups were *Bacteroides*, *Prevotella*, *Faecalibacterium*, *Blautia*, *Bifidobacterium*, *Collinsella*, *Agathobacter*, *Subdoligranulum*, *Fusicatenibacter*, and members of the family Lachnospiraceae.

**Figure 3:**
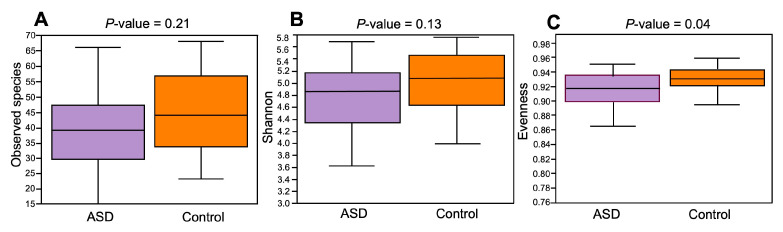
Alpha-diversity ecological analysis using observed species (**A**), Shannon (**B**), and Evenness indices (**C**). Legend: ASD—autism spectrum disorder group; control—control group.

The composition of the ASD and control groups was analyzed using α- and β-diversity. Concerning α-diversity, we determined the values of three diversity indices: observed species, Shannon index, and Evenness index (Figure 3). The values of these three indices were lower in the ASD group compared to the control group, but only the difference in the Evenness index was statistically significant (*p* = 0.04, Kruskal–Wallis’s test).

**Figure 4:**
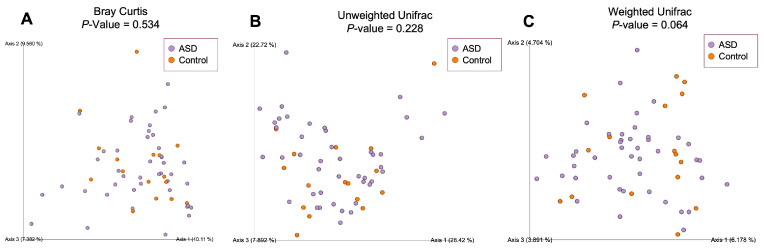
Beta-diversity ecological analysis using Bray–Curtis (**A**), unweighted Unifrac (**B**) and weighted Unifrac (**C**) distances. Legend: ASD—autism spectrum disorder group; control—control group.

To estimate compositional dissimilarity between the ASD and control group datasets, we then examined β-diversity (Figure 4). Three β-diversity metrics were applied: Bray–Curtis, unweighted Unifrac, and weighted Unifrac distances. PERMANOVA analysis showed no significant differences among the two studied groups (Bray–Curtis: *p* = 0.534, Pseudo-F = 0.99; unweighted Unifrac: *p* = 0.064, Pseudo-F = 1.64; weighted Unifrac: *p* = 0.228, Pseudo-F = 1.30).

### 3.3. Machine Learning Results

**Figure 5:**
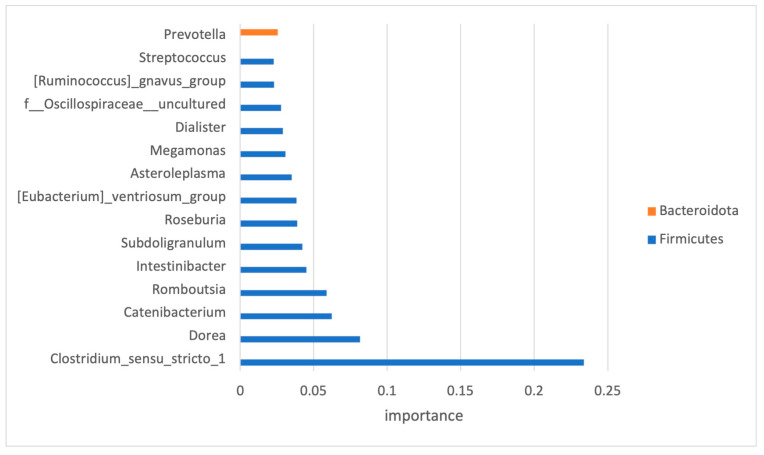
Significant OTUs selected by classification model analysis. The bars represent the importance scores.

As the number of features within the dataset was much higher than the number of samples, and to avoid the issue of high dimensionality, feature selection was performed. Only high-impact features (33 in total) were selected, and the top 15 and their impact on the classification are presented in Figure 5.

The original issue of the imbalanced dataset was overcome by applying the SMOTE, and the hyper-parameters of the estimators were tuned with the help of the GridSearchCV function. The original dataset contained groups of 44 and 16 samples, and after the upsampling, the groups were balanced (44 samples each).

As we dealt with a limited-size dataset (88 samples after SMOTE application), the training and testing data were split using the 5-fold cross-validation technique, allowing the most optimal balance between the training set and test fold. We performed 5-fold cross-validation both before and after applying SMOTE. This allowed us to compare the model’s performance with and without the synthetic samples. We concluded that the performance after applying SMOTE improves without overfitting (the performance on the validation set remained consistent with the test set). This indicated that the synthetic samples help the model to generalize without introducing bias.

Multiple cycles of the algorithm were run, and each time, one-fifth of the data were used for testing and the remaining data were used for training of the model (five random splits of data to 80% training samples and 20% testing samples).

The mean accuracy scores after running 5-fold cross-validation obtained at the genus level are shown in Table 3. The highest mean accuracy reached was 80%. The mean accuracy was calculated as the average accuracy after five runs of the cross-validation process had been applied.

### 3.4. Subject Characteristics

**Figure 6:**
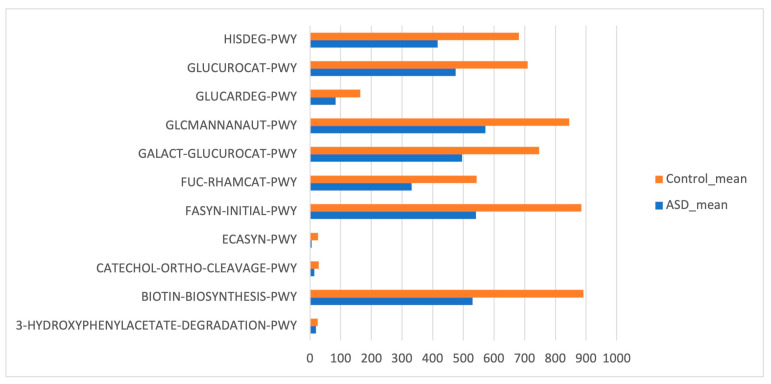
Kyoto Encyclopedia of Genes and Genomes (KEGG) biomarkers inferred from the whole set of OTUs of the patients with ASD and the control subjects.

Phylogenetic Investigation of Communities by Reconstruction of Unobserved States (PICRUSt) based on reference OTUs was applied to predict the abundances of the KEGG orthologs (KOs). There was a total of 454 functions related to both ASD and control samples. Few KEGG orthologs (Figure 6) showed significant differences in the gut microbiome of the ASD and control subjects (*p* value ≤ 0.05, Mann–Whitney U test).

**Figure 7:**
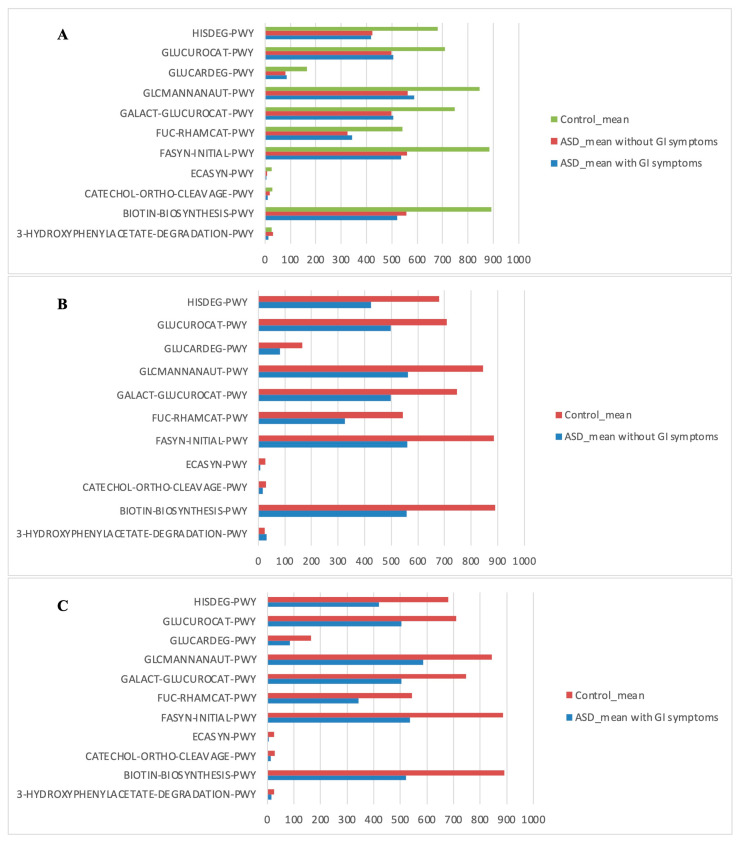
Kyoto Encyclopedia of Genes and Genomes (KEGG) biomarkers inferred from the whole set of OTUs of the patients with ASD grouped into the patients with and without GI symptoms versus the CTRL subjects. KO distribution for each group computed by the Mann–Whitney U test (*p* < 0.05). (**A**) ASD with GI symptoms versus ASD without GI symptoms and versus CTRLs; (**B**) ASD without GI symptoms versus CTRLs; (**C**) ASD with GI symptoms versus CTRLs.

The PICRUSt analysis was also segmented into ASD subgroups (with gastrointestinal issues and without gastrointestinal issues) compared to controls (Figure 7).

**Figure 8:**
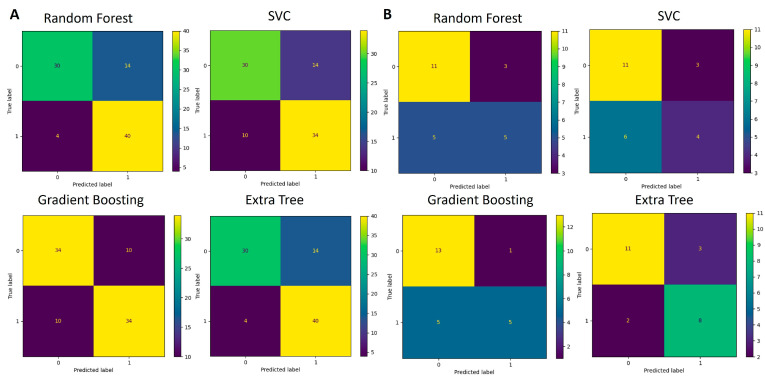
Confusion matrices for genus-based ML models. (**A**) Performance of four algorithms during the 5-fold cross-validation process (88 samples in total) used for model training and testing. (**B**) Performance of validation process where models developed in (**A**) were used for classification.

**Figure 9:**
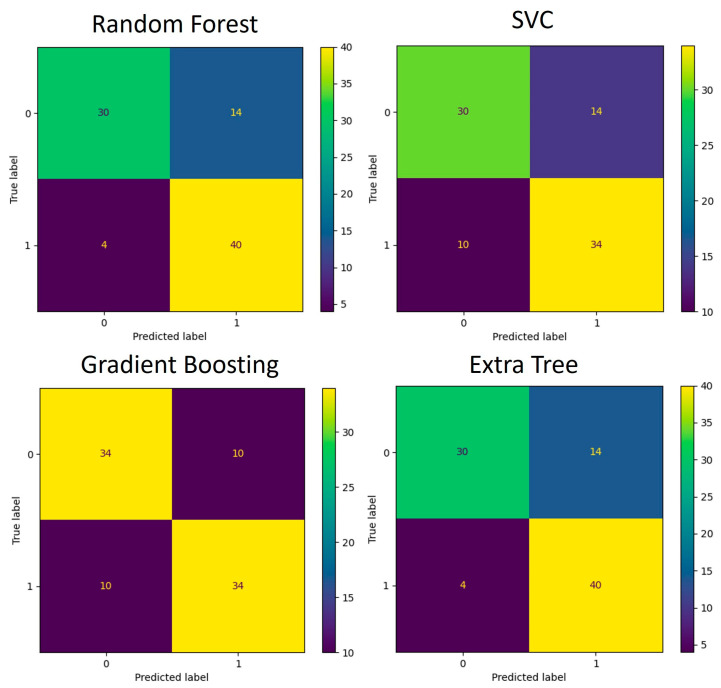
Confusion matrices for KEGG pathway-based ML models during the 5-fold cross-validation process (88 samples in total) used for model training and testing.

The functions identified as significant by Mann–Whitney U test (*p* < 0.05) were used for the development of an additional classification model like the one created for the taxa data. The training and testing data were split using the 5-fold cross-validation technique. Multiple runs of the algorithms were run, and each time, one-fifth of the data were used for testing and the remaining data were used for training of the model. The average accuracy scores obtained are listed in Table 4. The confusion matrices that depict the models’ performance are presented in Figure 8 and Figure 9.

### 3.5. Validation of Classification Model

The developed model was validated using an independent dataset. The validation dataset provided a total of 25 samples, 11 ASD and 14 control. The mean accuracy of prediction using the abundance of bacteria on the genus taxonomy level is summarized in Table 5. These data demonstrate that classifier performance is robust to the batch effect, though performance is somewhat lower.

## 4. Discussion

Early detection of ASD is associated with better outcomes as it enables early intervention. Gut microbiome sequences have been analyzed during the last decade, revealing that people with ASD have different gut microbiota than neurotypical people.

The purpose of this study was to sequence and analyze gut microbiome sequences of ASD patients and compare them to healthy controls and utilize them to develop a computational model that successfully distinguishes between these two groups.

The results of our study have, indeed, shown certain differences between the two observed groups. Unlike Son et al. [22], we observed a statistically important difference in the alpha-diversity of ASD and control groups reported by the Evenness metric. Beta-diversity analysis has not shown a significant difference between the two observed communities when tested with weighted Unifrac, unweighted Unifrac, and Bray–Curtis metrics.

The out-of-the-box statistical analysis was performed on the obtained taxonomic abundance information. Starting with the phylum level, seven phyla have been identified in the dataset: Firmicutes, Bacteroidetes, Proteobacteria, Actinobacteria, Verrucomicrobia, Cyanobacteria and Euryarchaeota. No significant difference has been found between ASD and control groups when non-parametric tests have been conducted, conflicting with the reports made by Finegold et al. [38]. Moreover, no significant difference has been found in the Firmicutes/Bacteroidetes ratio. The only statistically significant difference in abundances was obtained for the genus *Streptococcus* (and its complementary family-level bacteria), with it being decreased in the ASD group. This finding matches the findings reported in the previously published studies [28,39,40,41,42].

For the development of ML classifiers, we needed to designate the important features—the taxa (or predictive metabolic functions) that had a significant difference between the ASD and control groups. Out of 145 bacteria detected at the genus level, only 33 have been recognized as important for the classification modeling. Seventy-nine percent of the bacteria used for the classification modeling belonged to the Firmicutes phylum.

Some of the features, recognized as important, were previously implicated in ASD. One example is *Prevotella*, which was found enriched, albeit statistically non-significant, in the microbiome of children with ASD. Members of this bacterial genus have been associated with gut mucosa. Perturbation of the gut microbiome by *Prevotella* spp. has been shown to enhance host susceptibility to mucosal inflammation [43], a condition that has been linked to disruption of the intestinal epithelial barrier in ASD [44].

An increase in another group of mucosa-associated bacteria was observed in this study. This group was also among the selected high-impact features and corresponded to members of the genus *Clostridium* which were previously associated with functional abdominal pain in children with ASD [45]. Other selected high-impact features that were previously associated with ASD represented a minor portion of diversity. They included members of *Dorea*, *Dialister*, and *Streptococcus* that were enriched in the ASD dataset as observed in previous studies [46].

The tool used for predictive functions, PICRUSt [32], uses microbiome structure to predict the metabolic potential, and though it has limited accuracy, its power was strong enough for the development of ML models with significant predictive accuracy. The feature selection process that was conducted on the KEGG predictive functions returned only 14% of the total features to be used for the classification model.

Our ML-based data processing pipeline utilized four algorithms and developed two classification models for each. The dataset generated in this paper was used to develop the models. The models were trained and tested using a 5-fold cross-validation process.

When genus-level taxa were used for features, Random Forest and Extra Trees algorithms exhibited the highest accuracy. The Random Forest algorithm has been reported continuously to perform well on such microbiome-generated data [7]. Random Forest and Extra Trees outperform other algorithms in regard to the classification of microbiome data due to their capacity to handle high-dimensional, sparse, and noisy sets, as well as due to their robustness to class imbalance. The number of trees (n_estimators), tree depth (max_depth), minimum samples per leaf (min_samples_leaf), and class weighting (class_weight) were all important factors in enhancing their performance. Because of their capacity to average over several trees while focusing on the most informative features, these algorithms generally outperform other models in terms of microbiome data classification at the high-dimensional genera level. The sensitivity/recall is significantly higher than the specificity for tree-based algorithms (Random Forest and Extra Trees), which means that these algorithms are better at detecting ASD group samples but have a good number of control group samples identified as false positives. For the remaining two algorithms, the difference in those two metrics is less significant. Precision measures the percentage of true positives over all the samples that have been identified as positive and in our case is closely related to the accuracy metric. The F1 metric is the harmonic mean of recall and precision metrics and relates very closely to the accuracy metric results. In terms of exploitation of results for medical decision-making, this is an encouraging result as most ASD cases are correctly identified while some control samples are also flagged as ASD.

When KEGG pathways were used as features, the performance of all models dropped but the lowest accuracy was still 72%. The sensitivity/recall was again significantly higher than the specificity for tree-based algorithms (Random Forest and Extra Trees), which means that these algorithms were better at detecting ASD group samples but had a significant number of control group samples identified as false positives. On the other hand, the SVC algorithm classified the control group’s samples more accurately. The Gradient Boosting algorithm was equally successful for both groups. The precision metric was closely related to the accuracy metric except for the SVC algorithm due to significant differences between sensitivity and specificity metrics. The F1 metric relates closely to the accuracy metric except for the SVC algorithm.

The drop in classification performance when using KEGG pathways instead of genus-level taxa in microbiome studies can be attributed to several factors, despite the biological relevance of these functional features. To be precise, PICRUSt predicts functional potential rather than actual gene expression or activity, so there is a degree of uncertainty regarding whether the predicted pathways are truly active in the sample. The disconnect between the functional potential and actual functional activity may reduce the predictive power of KEGG pathways in distinguishing between classes. Moreover, many microbial species can contribute to the same KEGG pathway, leading to redundancy in the feature set. This redundancy makes it difficult for machine learning models to differentiate between classes based solely on functional profiles, as the same pathway may be associated with different taxa across samples, blurring the boundaries between groups.

When the performance of the ML models was tested on the validation set, whose data were independent of the model development, the ML algorithms kept their classification power, though it decreased. The highest accuracy was for the Extra Trees Classifier (79%). Interestingly, the sensitivity/recall metric was significantly lower than the specificity for all algorithms except the Extra Trees. This means that algorithms were better at detecting control group samples but had a significant number of ASD group samples identified as false negatives. The Extra Trees algorithm was equally successful for both groups. The precision and F1 metrics were also significantly different for all but the Extra Trees algorithm. These results suggest that the classification model, developed on a different dataset, performed better at identifying the control group’s samples than the ASD samples.

The data obtained by 16S rRNA sequencing technique distinguished ASD patients from the controls with around 80% accuracy. This value is promising for the introduction of computational methods in medical decision-making. Though this is a valuable contribution, further improvements can be made so that accuracy can be improved.

Overall, this study considered 60 samples, a relatively small number for data science. The mitigating factors are that the classification was binary and upsampling was performed due to an imbalance in the sample size of the two studied groups. More samples need to be considered to construct even more applicable and accurate results. In general, wider accessibility of ASD-associated metagenomics samples would potentially lead to the development of an even more precise classification model.

## 5. Conclusions

In this study, we investigated the microbiomes of children with ASD and neurotypical children in Bosnia and Herzegovina. Our focus was on examining the differences in the structure of gut microbiomes and highlighting patterns observed in previous similar studies. Key distinctions in the ASD group, when compared to the control group, that we discovered included decreased alpha-diversity, decreased abundance of members of genus *Streptococcus*, and underexpression of several KEGG orthologs.

Early intervention in ASD is linked with improved outcomes for the disorder. Consequently, this study aimed to construct a classification model utilizing gut microbiome data from ASD and neurotypical children. Our classification model demonstrated a high accuracy rate (80%) in predicting whether a given gut microbiome sample originated from a child with ASD. The validity of the classifier was confirmed using an independent dataset derived from Italian children.

To our knowledge, this is the first microbiome study in Bosnia and Herzegovina (and the wider region).

## Figures and Tables

**Table 1:** Metadata summary characteristics of the patients with ASD and controls.

Anamnestic Variables	ASD (*n* = 44)	Control (*n* = 16)
Age (years, mean (SD))	6.1 (1.71)	5.25 (1.77)
Gender (F = female, M = male)	37 (M)/7 (F)85%/16%	12 (M)/4 (F)75%/25%
Breastfeeding (percentage)	41/44 (93%)	15/16 (94%)
GI symptoms (percentage)	40/44 (90%)	2/16 (12.5%)
Speech disorder (percentage)	44/44 (100%)	3/16 (18.8%)

**Table 2:** Number of taxa identified at each taxonomic level.

Taxonomy Level	ASD	Control Group
Phylum	7	6
Class	15	13
Order	31	25
Family	51	41
Genus	134	95

**Table 3:** Classification results obtained with the dataset used for the development of the classification model. The basis for classification: genus-level taxa.

Classifier	Mean Accuracy	Sensitivity/Recall	Specificity	Precision	F1
Random Forest	80%	91%	68%	74%	82%
SVC	73%	77%	68%	71%	74%
Gradient Boosting	77%	77%	77%	77%	77%
Extra Trees	80%	91%	68%	74%	82%

**Table 4:** Classification results obtained with the dataset used for the development of the classification model. The basis for classification: KEGG pathways.

Classifier	Mean Accuracy	Sensitivity/Recall	Specificity	Precision	F1
Random Forest	78%	84%	73%	76%	80%
SVC	72%	52%	91%	85%	65%
Gradient Boosting	75%	75%	75%	75%	75%
Extra Trees	76%	82%	70%	73%	77%

**Table 5:** Classification results obtained with the dataset used for the validation of the classification model. The basis for classification: genus-level taxa.

Classifier	Mean Accuracy	Sensitivity/Recall	Specificity	Precision	F1
Random Forest	67%	50%	79%	63%	56%
SVC	63%	40%	79%	57%	47%
Gradient Boosting	75%	50%	93%	83%	63%
Extra Trees	79%	80%	79%	73%	76%

## Data Availability

The datasets generated and analyzed for this study can be found in the NCBI Sequence Read Archive under the BioProject accession number PRJNA1046003 (https://www.ncbi.nlm.nih.gov/bioproject/PRJNA1046003, accessed on 1 September 2024).
